# RNA Sequencing Reveals the Suitability of Cardiac Death Livers for Transplantation

**DOI:** 10.1155/2018/8217486

**Published:** 2018-11-01

**Authors:** Xinguo Chen, Zhongyang Shen, Xuyi Zhang, Jing Guo, Yuwen Hao, Li Cao, Yang Yue, Fengdong Wu, Yi Wang, Letian Wang, Sha Mao, Qing Zhang

**Affiliations:** ^1^Institute of Liver Transplantation, General Hospital of Chinese People's Armed Police Force, Beijing, China; ^2^Medical Department, General Hospital of Chinese People's Armed Police Force, Beijing, China; ^3^Disinfection Supply Department, General Hospital of Chinese People's Armed Police Force, Beijing, China

## Abstract

**Background:**

Organ transplantation is considered the best treatment for end-stage organ failure. However, the lack of available organs for transplantation and the increasing number of patients waiting for transplants are primary issues facing the transplant community. Thus, developing strategies to increase the number of donors, especially for liver transplantation, has become a priority. The use of organs acquired from donors who suffered cardiac related deaths has increased the pool of potential liver donors. However, donation after cardiac death (DCD) livers increases the risk of primary graft dysfunction.

**Methods:**

In the current study, we conducted transcriptome sequencing using livers from a DCD rat to assess the short-term feasibility and functional efficacy of DCD livers. RNA sequencing (RNAseq) data showed that the liver transcriptome varied greatly in rat livers subjected to 15 minutes of cardiac arrest.

**Results:**

The livers used in the current study had a significant loss of normal function before transplantation. Functional and network analyses consistently indicated that transcription and translation processes were inhibited after approximately 15 minutes of cardiac arrest. Moreover, the transcriptomic sequencing data provides significant insight for identifying functional genes and testing additional biological questions in DCD liver transplantation in future studies.

## 1. Introduction

The liver is a vital organ involved in many physiological and biochemical processes [1]. Transplant surgery is a reliable therapy for all types of advanced liver diseases. With gradual improvements in surgical methods of liver transplantation, the survival rate of liver failure patients has increased to 75% over the last five years [2]. Such improvements have heightened awareness regarding the therapeutic potential of liver transplantation, which has ultimately enhanced the demand for liver transplantation. However, the increasing number of recipients for liver transplantation and the decreased number of available donors are major hindrances in execution of the therapy.

Currently, transplant communities and governmental agencies are making collaborative efforts to increase the number of organ donors and to find a possible solution for the disparity between availability and need for transplantable organs. Considering the shortage of organs for donation, transplantation of livers from cardiac death donors (DCD) offers doctors and patients a potential way to meet the growing demand for liver transplantation [2]. In DCD liver transplantation, donor organ recovery is subject to determining ischemic injury after withdrawal of life support. Prolonged time between surgery and the withdrawal of life support is uniformly observed as harmful to the viability of DCD liver grafts due to poor perfusion and oxygenation, causing serious tissue damage that contributes to the obvious inferiority of liver transplantation [2]. Previous studies have reported that transplantation of livers obtained from prolonged DCD results in inferior outcomes compared to transplantation of livers donated immediately after deaths that are attributed to loss of brain function [3]. These inferior results are associated with high rates of biliary complications, as well as increased rates of primary nonfunction and hepatic artery thrombosis.

Although previous studies have demonstrated various risk factors for poor outcomes in DCD liver transplantation, the underlying mechanisms have not yet been determined. These studies had reservations regarding the use of liver grafts from DCD donors, so DCD liver transplantation cases have not increased as expected.

Next generation high-throughput RNA sequencing technology (RNAseq) provides a useful tool to generate transcriptomic resources to discover and determine putative genes and gene families. RNAseq provides a unique combination of transcriptome-wide coverage, sensitivity, and accuracy to comprehensively survey gene expression changes in one or more conditions [4]. RNAseq has been a powerful tool for studying global biological changes in various body organs. Global gene expression analysis of livers from a DCD rat provides a unique opportunity to study the functional deregulation in DCD livers at a molecular level. Considering the important role of the liver in metabolism and other functions of the human body, it is quite impossible to obtain dozens of human DCD livers for research. Therefore, animal models provided greater feasibility to identify possible underlying mechanisms of diseases with enigmatic pathogenesis. Rats are a more suitable animal model for studying liver metabolism and disorders compared to mice due to their physiological similarity with humans [5]. Thus, rats are common research* in vivo* models of different human diseases due to their size, short gestation period, and a mature genetic engineering technology for studying the rat genome.

In the present study, we conducted a timed-dependent RNAseq analysis using a DCD rat to understand the molecular mechanisms involved in the ineffectiveness of DCD liver transplantation. Our study provides a complete picture of the transcriptome variation in DCD liver samples, which will be helpful in filling the gap between theoretical and applicable knowledge of DCD livers used for transplantation. According to our results, there was a significant differential expression pattern of genes between 5 and 15 minutes after cardiac death, and we found that a series of genes related to metabolic biological processes show variable expression between time points, which could contribute to increased risks of transplantation. We also provide evidence that low temperatures could reduce the risk of damage to DCD livers for transplantation. However, more work is required to fully understand the suitability of DCD livers collected at low temperature for liver transplantation. In brief, a complete understanding of DCD liver functionality is necessary to develop therapeutics that will mitigate the risk of liver transplantation.

## 2. Materials and Methods

### 2.1. Sample Collection

Eight-week-old male brown Norway special pathogen-free (SPF) rats weighing between 350 and 400 g were purchased from Beijing Vital River Laboratory Animal Technology Company Limited in China. Rats (19 total) were randomly divided into seven groups (two or three rats per group). All rats were humanely sacrificed by CO_2_ asphyxiation and the left lateral lobes of the livers were collected at 0, 5, 15, 35, and 55 minutes after asphyxiation and stored at -80°C for further use. The collected samples were numbered as control and case groups S1-S4, respectively. To analyze the effect of temperature on DCD livers, two samples at 35 minutes and three samples at 75 minutes were collected at a low temperature (10°C) and named as case groups S5 and S6. All cell culture experiments or reproductive studies using animal models were approved by the Laboratory Animal Care and Use Committee of General Hospital of Armed Police Forces.

### 2.2. RNA Extraction and Sequencing

Total RNA was extracted from the cryopreserved rat liver samples using the TRIzol reagent according to the manufacturer's guidelines and treated with 20 units of RNase-Free DNase (Ambion, Shanghai, China) to remove the residual genomic DNA. RNA quality and quantity were analyzed using the Agilent 2100 Bioanalyzer (Agilent Technologies, Santa Clara, CA, USA) and Nanodrop 2000 (Thermo Scientific, Wilmington, DE, USA), respectively. RNA from each sample (5 *μ*g) was used to create the transcriptome libraries with IlluminaTruSeq™ RNA Sample Preparation Kit (Illumina, San Diego, CA, USA) following the manufacturer's guidelines. Sequencing was performed using the IlluminaHiSeq™2500 platform. Samples in groups S5 and S6 were kept under low temperature until sequencing was performed. Q20 was used as a quality control standard to filter the raw reads. Low quality reads were filtered, while the adaptors of high quality reads were removed, and the clean reads were aligned with the rat genome (Rnor_6.0) using HISAT [2]. No more than two mismatches were allowed in the alignment.

### 2.3. Differential Expression and GO Enrichment Analysis

Gene expression was determined using the FPKM method [2]. Differentially expressed genes (DEGs) were identified using DESeq2 [6] with significance set at* P* = 0.1, which was corrected applying the Benjamini and Hochberg False Discovery Rate (FDR) method. Gene ontology association data were downloaded from the gene ontology (GO) database (http://www.geneontology.org/) and enrichment analysis of biological processes was conducted using topGO. FDR correction was used to identify significantly enriched GO terms even when the FDR was less than 0.05. All statistical analyses were performed using R 3.0.0 (www.bioconductor.org).

### 2.4. Pathway and Network Analysis

Significant DEGs were imported into Ingenuity Pathways Analysis (IPA) software (Ingenuity Systems, Mountain View, CA, USA) to query possible biochemical, molecular, and biological functions. Genes were categorically organized according to disease, functions, and canonically available pathways and were ranked by* z*-score and* P* value, respectively. Network analysis of DEGs was also performed using IPA (Ingenuity® Systems, www.ingenuity.com) [7].

## 3. Results

**Figure 1:**
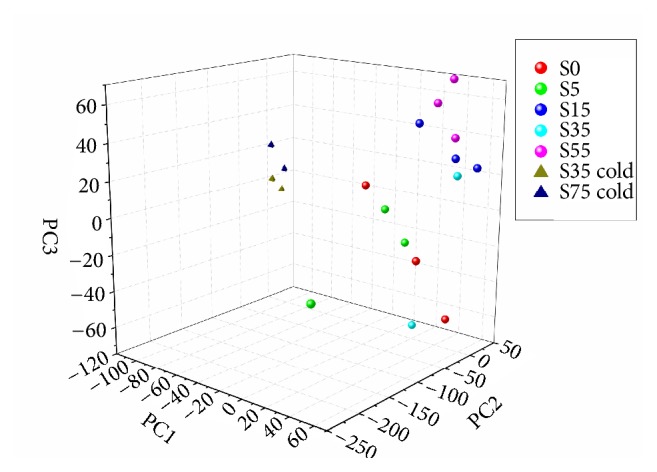
Principal component analysis (PCA) of the large-scale structure in gene expression data using the first three PCs. Different colors denote samples that were sequenced at different time points.

**Figure 2:**
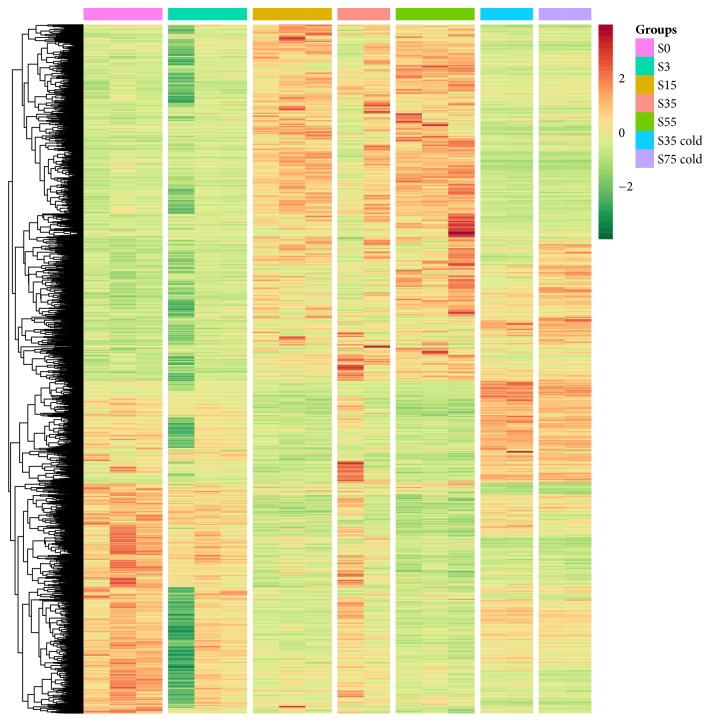
Hierarchical clustering analysis (HCA) using the expression data of all the DEGs (columns were sorted by sample information instead of being clustered). DEGs are represented as a combination of the individual DEGs in each comparison, including S0_vs_S5, S5_vs_S15, S15_vs_S35, S35_vs_S55, and S55_vs_S75. The heatmap based on the fpm (fragments of transcript per million mapped reads) was plotted for each scaled row.

A total of 526,154,477 clean reads from all 19 samples were obtained by high-throughput RNAseq (Supplementary Sheet 1 and Supplementary Sheet 2). To evaluate the consistent and specific characteristics of the transcription profiles under different conditions, principal component analysis (PCA) and unsupervised hierarchal cluster analysis (HCA) were conducted. In the three-dimensional (3D) space scatter plot of all samples, three obvious sample clusters were obtained: cluster 1, including groups S0-S5; cluster 2, including S15-S55; and cluster 3, including S35 cold and S75 cold. The samples in the S35 group had strong individual variability, which indicates that the liver suffered drastic changes (Figure 1). HCA results confirmed that the gene expression profiles, as well as the pattern of these profiles, were similar within each DCD group (Figure 2). Compared to the control samples (S0), a similar transcription pattern was observed in the S1 group, which indicates that the DCD livers were not affected within 5 minutes. The transcription level from group S15 at 15 minutes began to show differences compared to the control group, group S35 at 35 minutes, and group S55 at 55 minutes (Figure 2), which are consistent with the biopsy results (Supplemental Figures 1–3).

Moreover, we determined that expression of the transcripts from samples in cluster 3 (groups S35 cold and S75 cold) had a distinct expression pattern compared to cluster 2 (S35 group and S55 group). Low temperatures can slow organ decay, and therefore temperature could significantly affect DCD liver function. The high resemblance of gene expression profiles among all of the samples indicates that a proper low temperature could effectively change liver metabolism and preserve function of the DCD livers before transplantation.

**Figure 3:**
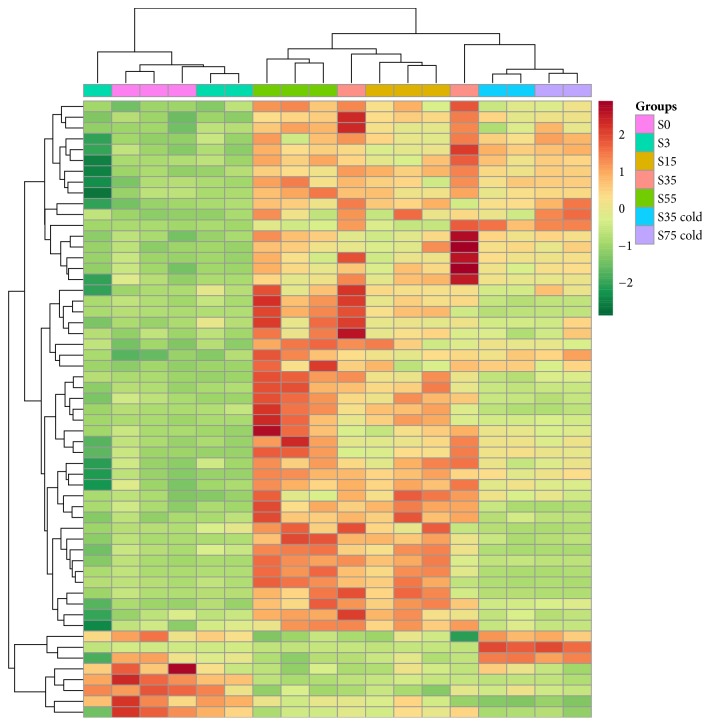
Hierarchical clustering analysis (HCA) of the DEGs between S0 and all other samples showing the different expression pattern of each group at different time points (columns were sorted by sample information instead of being clustered).

Based on previous PCA and HCA analyses, we compared variations in gene expression of three clusters. DEGs were identified between cluster 2 and cluster 1 and cluster 3 and cluster 1, respectively. Candidate genes for further analysis were then selected from the list of significant DEGs (FDR < 0.1) between the DCD clusters. The DEGs summarized in Supplementary Table 1 were further studied. In total, 57 significant DEGs were identified between cluster 2 and cluster 1, in which 8 genes were downregulated and 49 genes were upregulated. Similarly, 45 significant DEGs were identified between cluster 3 and cluster 1, among which 4 genes were downregulated and 41 were upregulated. Based on the DEGs, we further verified the expression pattern of these samples and all samples showed a similar expression pattern as determined by the comparison between S0 and all other samples (Figure 3), which indicates the reliability of our data. We also observed that a set of genes that were consistently active in the cold collected samples may be promising candidates for further study.

Among the significant DEGs, some have already been reported to exert an important effect on liver functions. For instance, it has been reported that* Nup62 *is important for nuclear production exchange and* Hsd17b10 *is an essential enzyme for liver metabolism [2]. The* YIPF* protein family showed a broad effect on protein modification [2].

To further explore the DEGs in DCD livers, biological process enrichment analysis was performed to determine global functional variations. We performed GO enrichment analysis using the DEGs obtained from the different CDC liver clusters. Significant DEGs identified from comparison of cluster 2 and cluster 1 and comparison of cluster 3 and cluster 1 were used in GO enrichment analysis, respectively. Enrichment results demonstrate that 122 and 198 GO biological processes were significantly enriched (Supplementary Tables 2 and 3). The results of the two comparisons identified 70 overlapped GO terms, which could not be predicted by chance (chi-square test t,* P *< 2.00E-16). Among these GO terms, we found a large number of significantly enriched metabolic related biological processes (FDR < 0.05), such as “cellular metabolic process” (GO:0050877, FDR = 2.03E-28, Supplementary Table 2), “organic substance metabolic process” (GO:0071704, FDR = 8.79E-23, Supplementary Table 2), and “primary metabolic process” (GO:0044238, FDR = 2.90E-19, Supplementary Table 2).

### 3.1. Complex Gene Networks Are Involved in Liver Corruption over Time

**Figure 4:**
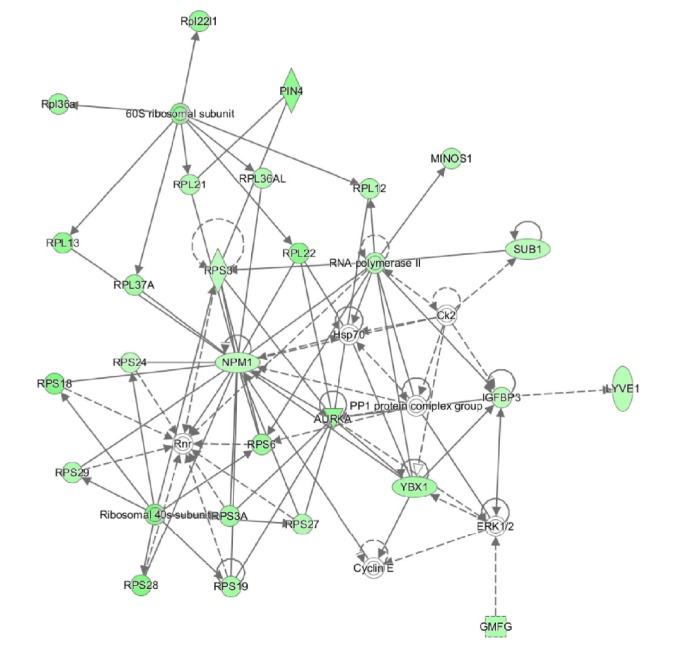
Regulation network of DEGs and other small molecules in IPA analysis. Green represents downregulated gene expression. Rhombi, ovals, squares, rectangles, dash squares, dash rectangles, trapezoids, circles, double circles, triangles, and inverted triangles represent enzyme, transcription regulator, cytokine, G-protein coupled receptor, growth factor, ion channel, transporter, other, complex/group, phosphatase, and kinase, respectively. Solid arrows and dashed arrows represent direct and indirect activation, respectively.

To gain insight into how DEGs function in the early time point of DCD livers, we further constructed a systematic regulation network by recruiting the functional proteins, genes, and other miRNAs that interact with the identified DEGs based on the IPA platform, to build a regulatory relationship. DEGs representing a 2-fold ratio compared to the control group were inputted into the IPA platform [7] to build a network analysis. IPA computed a score for each network according to the fit of the set of supplied DEGs. These scores, derived from* P* values, indicated the likelihood that DEGs belong to a network versus those obtained by chance. A score above 2 indicated > 99% confidence that a candidate gene network was not generated by chance [2]. The networks with a score above 20 were selected for further analysis. These networks included two types of genes, DEGs and genes directly connected with DGEs. Based on the IPA platform, five networks were obtained as presented in Table 1 using the DEGs identified between cluster 2 and cluster 1. The network with the highest IPA score is presented in Figure 4. Results of IPA analysis showed that this network was significantly related to “Cell Death and Survival” and “Organismal Injury and Abnormalities”, which may be highly related to the condition of DCD livers after a prolonged procurement period. In this network, we found that most of the genes were downregulated. RNA polymerase II, one of the most important enzymes in transcription and the main regulator in this network, was significantly downregulated in the S15-S55 groups. Moreover, another central gene in this network,* NPM1 *(nucleophosmin 1), which can inhibit proliferation and induce cell apoptosis [8], was significantly downregulated. Similarly, a total of 25 networks were identified based on the DEGs detected in cluster 3 and cluster 1. The network list showed that the network with the highest IPA score was significantly related to “Cell Death and Survival” and “Organismal Injury and Abnormalities”, which was consistent with the above mentioned results. All of these IPA networks are shown in Supplementary Table 4.

## 4. Discussion

Regardless of the efforts and advancements made in liver transplantation, it is crucial to assess the condition of the donated liver to optimize the use of marginal livers and to improve the clinical outcomes in the recipient [9]. Currently, the mechanism underlying loss of function in DCD livers remains poorly understood. A series of factors, such as metabolic capacity and cellular injury, can significantly affect liver function.

No transcriptome data is currently available to assess DCD liver quality for transplantation. Most studies rely on differential gene expression at a fixed time point, with very little focus on gene expression studies at varied time points during the entire process of liver harvesting. As such, there is not much reliability in distinguishing DCD liver quality, as different biological situations affect different functions that will lead to a cyclical, sustained, or peaked responses [2]. In this study, we conducted a time series RNAseq analysis of livers using a rat DCD model, which is the first study to provide a complete picture of transcriptome fluctuation in DCD livers. Our findings provide insight into the deep gap between theory and application of DCD livers for transplantation.

Dramatic changes in mRNA levels may produce substantial fluctuations in biology, which can facilitate rapid translational repression and/or degradation of hundreds of other genes. The RNAseq data in the current study provides a chance to visualize time series global gene expression changes in DCD livers. Using unsupervised clustering of the expression profiles at different time points, we visualized dramatic changes in gene expression between 15 minutes and > 15 minutes after cardiac death, which may be an important indicator of functional dysregulation [9, 10]. However, the expression profiles of the samples kept under low temperature were quite similar compared to the early time DCD liver expression profiles, even after a relatively longer time (35 and 75 minutes after cardiac death), which suggests that low temperature treatment could be a suitable mechanism to maintain liver function during the transplantation process.

Global DEG analysis revealed that some important genes, such as* YIPF*, lost their function shortly following cardiac death. The liver is an important organ in controlling and maintaining metabolism and thus plays a major role in functional protein production. It has already been demonstrated that maintenance of* YIPF* is important for preserving the Golgi apparatus for further protein modification [2]. However, our results showed that* YIPF* was significantly downregulated in DCD livers after 15 minutes of cardiac death, which could underlie the dysfunction of DCD livers. Thus, downregulation of different genes could be a primary risk factor in liver transplantation. Moreover, a number of pathways, such as cell cycle, metabolism, and kinase signaling, could be relevant to the activity response of DCD livers [9–11]. Enrichment analysis showed that DEGs between high risk group samples (DCD liver samples > 15 minutes of cardiac death) were involved in a series of metabolic biological processes. Specifically, “organic substance metabolic process” and “primary metabolic process” were significantly enriched in DCD livers. These results suggest that a huge energy consumption is necessary to maintain activity of DCD liver cells. Therefore, liver cells may lose their function if energy consumption is disturbed or dysregulated, and risk factors for transplantation of DCD livers increase with prolonged harvest time. However, analysis of the transcriptome with increasing time provides us an easy way to distinguish the risk level of candidate DCD livers.

There are some limitations in our study. Specifically, we observed only subtle differences in gene expression between the high and low risk DCD groups. Although we carefully analyzed the time series transcriptome expression patterns, we could not analyze more time points due to funding constraints. Thus, it is quite possible that our analysis might have neglected potential key regulators. Therefore, in order to gain a better understanding of the poorly defined mechanisms of DCD livers, further work is required.

In summary, transcriptome expression patterns could be used to clinically ascertain DCD liver quality and outcomes. However, more studies are required to optimize the selection of suitable DCD livers for transplantation.

## Figures and Tables

**Table 1:** Network results obtained using the IPA platform.

**ID**	**Score**	**Focus Molecules**	**Diseases & Functions**	**Molecules in Network**
1	60	26	Cancer, Cell Death and Survival, Organismal Injury and Abnormalities	60S ribosomal subunit, AURKA, Ck2, Cyclin E, ERK1/2, GMFG, Hsp70, IGFBP3, LYVE1, MINOS1, NPM1, PIN4, PP1 protein complex group, Ribosomal 40s subunit, RNA polymerase II, Rnr, RPL12, RPL13, RPL21, RPL22, Rpl22l1, Rpl36a, RPL36AL, RPL37A, RPS3, RPS6, RPS18, RPS19, RPS24, RPS27, RPS28, RPS29, RPS3A, SUB1, YBX1
2	32	16	Nucleic Acid Metabolism, Small Molecule Biochemistry, Energy Production	adenosine-tetraphosphatase, ANAPC13, ATP synthase, ATP5D, ATP5F1, ATP5J, ATP5J2, Atp5k, ATP5L, ATP5S, C20orf24, C8orf59, COLEC11, Cox7c, DDX3X, DNAJC28, EED, EIF3J, ENY2, ESR1, F0 ATP synthase, FOXRED1, GNGT2, HSPA5, LRRK2, LSM5, MT-ATP6, mt-Atp8, MYL9, NXF1, RICTOR, Snrpe, SRPK2, USMG5, YIPF2
3	24	13	Organismal Injury and Abnormalities, Cellular Development, Hematological System Development and Function	Akt, APOA2, COL3A1, Collagen type I, Collagen(s), ERK, FLI1, GJA1, HDL, Hsp90, IgG, IL1, Insulin, Jnk, LDL, LOC100365810/Rps17, Mapk, Mmp, NDUFA1, NME1, ORM1, P38 MAPK, Pdgf (complex), PDGF BB, PI3K (complex), Pka, Pkc(s), PPIA, PPIG, Ras, SPARC, SPARCL1, Tgf beta, TMSB10/TMSB4X, Vegf
4	24	13	Cell-To-Cell Signaling and Interaction, Connective Tissue Development and Function, Cancer	ACAD10, ADH4, ALAD, APP, ATP6V0E1, CCDC80, CCK, CCND1, CDKN1A, CES3, CHURC1, GATM, Gsta4, Gstt1, HADH, HSD17B10, IL4, IL6, Ins1, IRF2BP2, IVD, LGALS3, Marcks, Mt1m/Mt2A, NPHP1, PAQR9, PDHX, PPARA, RNASE4, RTN4RL2, Spink1/Spink3, Tomm5, TOMM40, VCAN, ZBED5
5	20	11	DNA Replication, Recombination, and Repair, RNA Post-Transcriptional Modification, Connective Tissue Disorders	Actin, caspase, CD3, Cg, CLDN11, ELP5, ELP6, FSH, GDE1, Histone h3, Histone h4, HLA-DQA1, HLA-DQA2, HLA-DQB3, HLA-DRB3, HLA-DRB4, HLA-DRB5, HNRNPA1, HNRNPC, LARP1B, MHC Class II (complex), MHC II, MHC II-*β*, Mhc2 Alpha, MRPS18C, NFkB (complex), NFX1, NUP62, p85 (pik3r), PCBD2, POLR2K, RFXAP, RPL12, SAP18, Sin3

## Data Availability

The datasets generated in the current study are available in the NCBI Sequence Read Archive (Accession no. SRP155446).

## Supplementary Materials

Supplementary Materials Supplementary Sheet 1: the sample information for all the sample used in this study, including the accession number and data size. Supplementary Sheet 2: the raw count of each gene for all the samples sequenced in this study. The raw count of each gene was estimated by ht-seq with default parameters. Supplemental Figure 1. Pathological section of rat liver after 15 minutes of death. Supplemental Figure 2. Pathological section of rat liver after 35 minutes of death. Supplemental Figure 3. Pathological section of rat liver after 55 minutes of death. Supplementary Table 1. Selected differentially expressed genes. Supplementary Table 2. Biological process enrichment results using differentially expressed genes between cluster 2 and cluster 1. Supplementary Table 3. Biological process enrichment results using differentially expressed genes between cluster 3 and cluster 1. Supplementary Table 4. Network results using the IPA platform.
