# Supplementary material for: RNA Sequencing Reveals the Suitability of Cardiac Death Livers for Transplantation
Source: Biomed Res Int. 2018 Nov 1;2018:8217486. doi: 10.1155/2018/8217486 (PMC6241343; doi:10.1155/2018/8217486)
Supplement: Supplementary Materials — Supplementary Sheet 1: the sample information for all the sample used in this study, including the accession number and data size. Supplementary Sheet 2: the raw count of each gene for all the samples sequenced in this study. The raw count of each gene was estimated by ht-seq with default parameters. Supplemental Figure 1. Pathological section of rat liver after 15 minutes of death. Supplemental Figure 2. Pathological section of rat liver after 35 minutes of death. Supplemental Figure 3. Pathological section of rat liver after 55 minutes of death. Supplementary Table 1. Selected differentially expressed genes. Supplementary Table 2. Biological process enrichment results using differentially expressed genes between cluster 2 and cluster 1. Supplementary Table 3. Biological process enrichment results using differentially expressed genes between cluster 3 and cluster 1. Supplementary Table 4. Network results using the IPA platform. [file 8217486.f1.zip › 8217486.f1/8217486.v3 (1) 24.pdf]

Supplemental figures

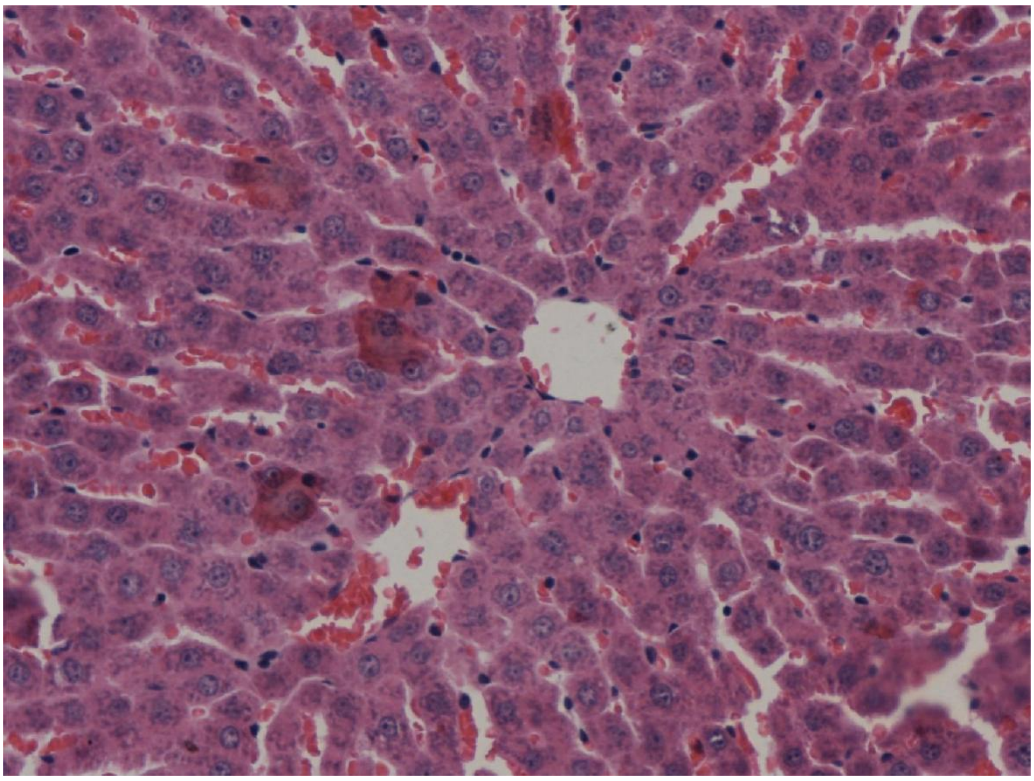

Supplemental Figure 1. Pathological section of rat liver after 15 minutes of death.

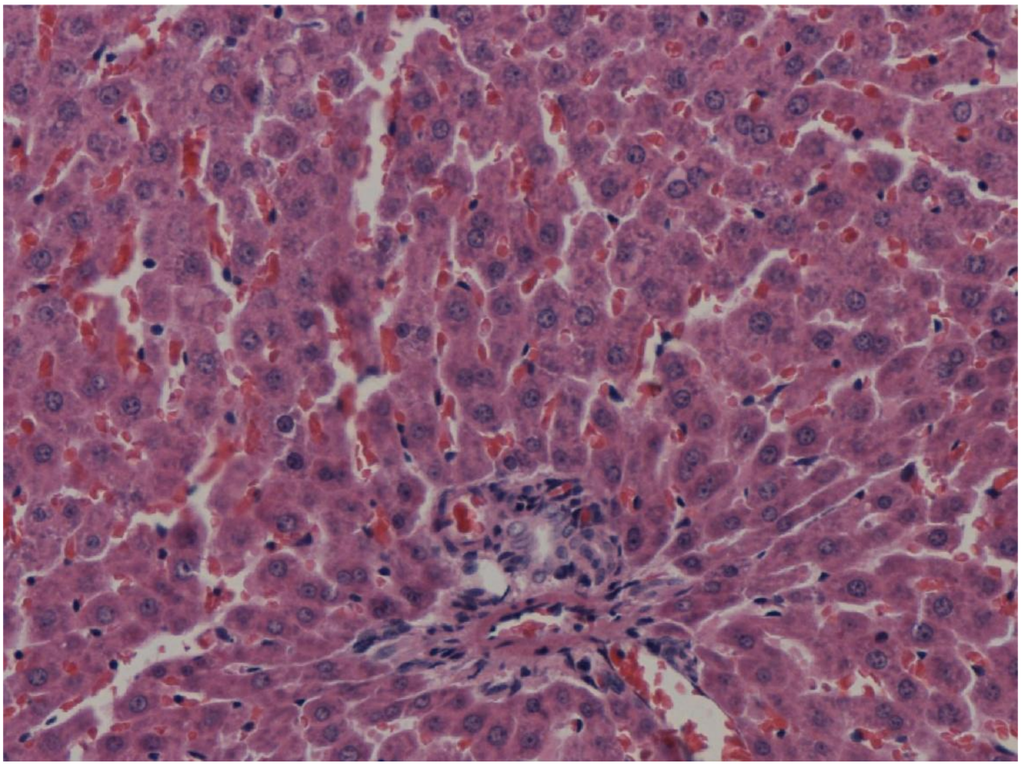

Supplemental Figure 2. Pathological section of rat liver after 35 minutes of death.

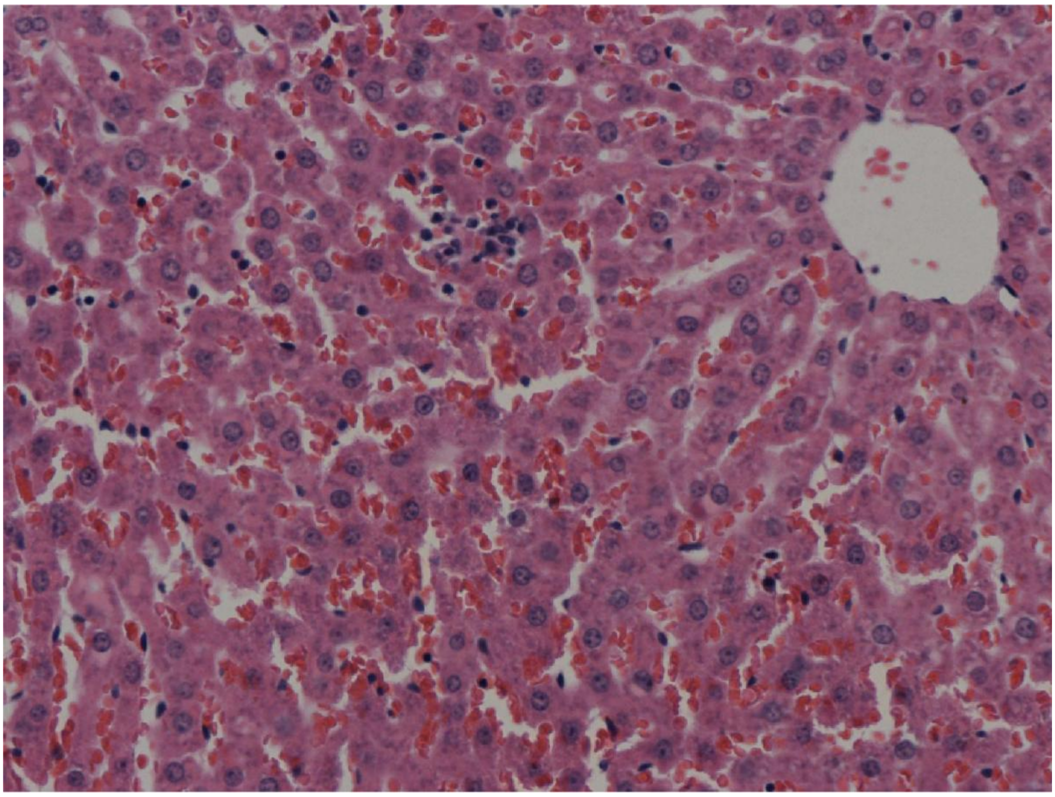

Supplemental Figure 3. Pathological section of rat liver after 55 minutes of death.

Supplementary Table 1. Differentially expressed genes selected.

Characteristics	cluster2 vs cluster1			cluster3 vs cluster1		
ID	FC	P	FDR	FC	P	FDR
ENSRNOG00000008459	3.16	8.55E-07	3.77E-05	2.31	9.19E-04	2.91E-03
ENSRNOG00000050051	4.27	1.43E-06	3.77E-05	2.25	8.28E-04	2.78E-03
ENSRNOG00000010732	3.60	2.64E-06	3.77E-05	1.17	3.55E-01	3.89E-01
ENSRNOG00000011104	6.94	5.33E-06	5.83E-05	2.14	8.13E-03	1.45E-02
ENSRNOG00000011494	6.67	6.14E-06	5.83E-05	1.91	4.17E-02	5.80E-02
ENSRNOG00000016220	2.78	1.14E-05	8.15E-05	1.82	1.65E-02	2.69E-02
ENSRNOG00000004479	6.54	1.65E-05	9.62E-05	1.82	5.25E-02	6.80E-02
ENSRNOG00000046349	4.91	1.98E-05	9.62E-05	1.73	4.83E-02	6.55E-02
ENSRNOG00000015335	6.06	1.99E-05	9.62E-05	1.15	6.04E-01	6.50E-01
ENSRNOG00000004681	2.48	2.02E-05	9.62E-05	2.05	5.46E-03	1.00E-02
ENSRNOG00000012840	1.83	2.81E-05	1.23E-04	1.43	3.69E-04	1.74E-03
ENSRNOG00000000805	3.05	3.10E-05	1.26E-04	3.22	1.04E-04	1.04E-03
ENSRNOG00000002052	2.05	4.64E-05	1.69E-04	1.95	1.09E-04	1.04E-03
ENSRNOG00000047796	3.40	4.75E-05	1.69E-04	1.58	2.81E-03	5.72E-03
ENSRNOG00000018426	2.53	5.53E-05	1.85E-04	1.95	3.53E-05	9.86E-04
ENSRNOG00000024967	3.27	5.95E-05	1.88E-04	0.93	8.05E-01	8.35E-01
ENSRNOG00000019838	3.20	7.16E-05	2.15E-04	1.59	1.13E-03	2.93E-03
ENSRNOG00000038902	2.72	8.31E-05	2.37E-04	1.71	3.37E-02	4.93E-02
ENSRNOG00000008373	2.22	9.50E-05	2.58E-04	1.91	3.24E-03	6.38E-03
ENSRNOG00000027864	4.55	1.23E-04	3.19E-04	1.42	1.69E-01	1.97E-01
ENSRNOG00000034237	13.48	1.49E-04	3.53E-04	1.39	6.78E-01	7.15E-01
ENSRNOG00000000579	2.50	1.54E-04	3.53E-04	2.28	6.61E-04	2.36E-03
ENSRNOG00000015093	2.41	1.63E-04	3.53E-04	1.90	1.11E-03	2.93E-03
ENSRNOG00000028505	7.92	1.67E-04	3.53E-04	1.65	2.56E-01	2.86E-01
ENSRNOG00000003357	2.54	1.67E-04	3.53E-04	1.46	1.68E-01	1.97E-01
ENSRNOG00000002178	2.33	2.32E-04	4.73E-04	1.58	2.33E-04	1.48E-03
ENSRNOG00000031641	16.70	2.59E-04	5.08E-04	3.71	5.44E-02	6.90E-02
ENSRNOG00000048109	5.60	2.78E-04	5.29E-04	2.59	3.78E-06	2.15E-04
ENSRNOG00000033215	2.86	2.96E-04	5.43E-04	3.00	6.92E-05	9.86E-04
ENSRNOG00000020246	2.15	3.12E-04	5.55E-04	1.34	2.12E-01	2.42E-01
ENSRNOG00000026902	1.63	3.43E-04	5.82E-04	1.45	1.04E-02	1.80E-02
ENSRNOG00000007673	2.27	4.03E-04	6.54E-04	2.03	2.71E-03	5.72E-03
ENSRNOG00000032803	3.98	4.13E-04	6.54E-04	2.73	3.96E-04	1.74E-03
ENSRNOG00000013410	3.35	5.20E-04	7.80E-04	1.30	5.13E-02	6.80E-02
ENSRNOG00000031127	3.55	5.65E-04	8.25E-04	2.29	2.62E-03	5.72E-03
ENSRNOG00000007336	2.89	6.38E-04	9.09E-04	2.89	1.08E-03	2.93E-03
ENSRNOG00000025764	3.91	9.47E-04	1.32E-03	3.02	6.01E-04	2.36E-03
ENSRNOG00000006108	3.49	1.39E-03	1.84E-03	3.63	2.53E-03	5.72E-03
ENSRNOG00000005203	2.41	1.43E-03	1.86E-03	1.75	3.53E-03	6.70E-03

ENSRNOG00000023385	2.58	1.66E-03	2.11E-03	1.81	1.94E-02	3.08E-02
ENSRNOG00000000451	2.45	2.03E-03	2.51E-03	2.09	2.25E-02	3.46E-02
ENSRNOG00000050000	4.10	2.25E-03	2.73E-03	2.43	1.54E-02	2.58E-02
ENSRNOG00000031022	2.19	2.32E-03	2.75E-03	1.81	9.75E-04	2.93E-03
ENSRNOG00000051284	7.21	2.54E-03	2.96E-03	5.14	3.28E-04	1.74E-03
ENSRNOG00000042886	4.57	2.59E-03	2.96E-03	1.12	8.48E-01	8.63E-01
ENSRNOG00000061438	5.79	3.33E-03	3.73E-03	3.16	1.46E-04	1.05E-03
ENSRNOG00000019106	3.36	4.61E-03	5.05E-03	2.39	1.62E-03	4.01E-03
ENSRNOG00000049829	3.71	4.95E-03	5.32E-03	2.35	2.35E-02	3.52E-02
ENSRNOG00000020296	5.99	1.80E-02	1.86E-02	10.04	6.25E-04	2.36E-03
ENSRNOG00000048733	0.60	3.47E-04	5.82E-04	0.98	8.63E-01	8.63E-01
ENSRNOG00000003049	0.23	1.11E-03	1.50E-03	10.37	2.75E-03	5.72E-03
ENSRNOG00000022043	0.61	8.88E-02	8.88E-02	1.53	1.22E-01	1.47E-01
ENSRNOG00000049900	0.54	5.12E-03	5.41E-03	0.69	1.20E-01	1.47E-01
ENSRNOG00000000918	0.29	2.20E-06	3.77E-05	0.32	1.48E-04	1.05E-03
ENSRNOG00000050445	0.44	8.95E-06	7.29E-05	0.54	3.78E-04	1.74E-03
ENSRNOG00000021513	0.57	5.81E-02	5.91E-02	0.36	3.95E-02	5.63E-02
ENSRNOG00000018935	0.56	5.05E-04	7.78E-04	0.27	5.96E-05	9.86E-04

Supplementary Table 2. Biological process enrichment results using differentially expressed genes between cluster 2 and cluster 1 samples.

GO ID	GO biological process complete	Enrichment P-value	FDR
GO:0050906	detection of stimulus involved in sensory perception	1.98E-33	2.42E-31
GO:0050907	detection of chemical stimulus involved in sensory perception	5.56E-33	6.73E-31
GO:0007606	sensory perception of chemical stimulus	4.11E-32	4.93E-30
GO:0007186	G-protein coupled receptor signaling pathway	9.37E-32	1.12E-29
GO:0009593	detection of chemical stimulus	3.47E-31	4.09E-29
GO:0007600	sensory perception	3.85E-31	4.50E-29
GO:0044237	cellular metabolic process	1.75E-30	2.03E-28
GO:0051606	detection of stimulus	4.39E-29	5.05E-27
GO:0050877	neurological system process	4.42E-29	5.05E-27
GO:0008152	metabolic process	3.94E-28	4.45E-26
GO:0071704	organic substance metabolic process	7.85E-25	8.79E-23
GO:0008150	biological_process	2.64E-22	2.93E-20
GO:0044238	primary metabolic process	2.64E-21	2.90E-19
GO:0003008	system process	2.11E-20	2.30E-18
GO:0006807	nitrogen compound metabolic process	2.80E-17	3.02E-15
GO:1901564	organonitrogen compound metabolic process	1.62E-16	1.73E-14
GO:0034641	cellular nitrogen compound metabolic process	2.45E-15	2.60E-13
GO:0009987	cellular process	7.58E-14	7.96E-12
GO:0044710	single-organism metabolic process	1.44E-12	1.50E-10
GO:0009058	biosynthetic process	2.33E-12	2.40E-10
GO:0044260	cellular macromolecule metabolic process	8.90E-12	9.08E-10
GO:1901576	organic substance biosynthetic process	1.12E-11	1.13E-09
GO:0044249	cellular biosynthetic process	5.28E-11	5.28E-09
GO:1901566	organonitrogen compound biosynthetic process	4.52E-10	4.47E-08
GO:1901360	organic cyclic compound	5.56E-10	5.45E-08

	metabolic process		
GO:0044281	small molecule metabolic process	1.75E-09	1.70E-07
GO:0006518	peptide metabolic process	2.27E-09	2.18E-07
GO:0043603	cellular amide metabolic process	3.25E-09	3.09E-07
GO:0010467	gene expression	3.68E-09	3.46E-07
GO:0044267	cellular protein metabolic process	4.99E-09	4.64E-07
GO:0071840	cellular component organization or biogenesis	6.24E-09	5.74E-07
GO:0044271	cellular nitrogen compound biosynthetic process	8.97E-09	8.16E-07
GO:0006412	translation	1.41E-08	1.27E-06
GO:0043170	macromolecule metabolic process	1.52E-08	1.35E-06
GO:0043604	amide biosynthetic process	2.09E-08	1.84E-06
GO:0043043	peptide biosynthetic process	2.56E-08	2.23E-06
GO:0044085	cellular component biogenesis	3.17E-08	2.73E-06
GO:0051234	establishment of localization	3.29E-08	2.80E-06
GO:0006810	transport	3.86E-08	3.24E-06
GO:0006725	cellular aromatic compound metabolic process	5.23E-08	4.34E-06
GO:0046483	heterocycle metabolic process	2.55E-07	2.09E-05
GO:0006139	nucleobase-containing compound metabolic process	2.60E-06	2.11E-04
GO:0022613	ribonucleoprotein complex biogenesis	4.97E-06	3.98E-04
GO:0016043	cellular component organization	8.41E-06	6.64E-04
GO:0006082	organic acid metabolic process	1.43E-05	1.12E-03
GO:0051179	localization	1.58E-05	1.22E-03
GO:0019538	protein metabolic process	1.86E-05	1.41E-03
GO:0010033	response to organic substance	2.16E-05	1.62E-03
GO:0044711	single-organism biosynthetic process	3.02E-05	2.23E-03
GO:0023052	signaling	4.39E-05	3.20E-03
GO:0044700	single organism signaling	4.80E-05	3.46E-03
GO:0006396	RNA processing	6.58E-05	4.67E-03
GO:0016070	RNA metabolic process	9.03E-05	6.32E-03
GO:0019637	organophosphate metabolic process	9.28E-05	6.40E-03
GO:0043933	macromolecular complex subunit organization	9.46E-05	6.43E-03
GO:0007165	signal transduction	1.16E-04	7.77E-03
GO:0009059	macromolecule biosynthetic process	1.20E-04	7.92E-03
GO:0007154	cell communication	1.28E-04	8.32E-03

GO:0046128	purine ribonucleoside metabolic process	1.51E-04	9.66E-03
GO:1901575	organic substance catabolic process	1.54E-04	9.70E-03
GO:0034645	cellular macromolecule biosynthetic process	1.82E-04	1.13E-02
GO:0009056	catabolic process	2.02E-04	1.23E-02
GO:0042278	purine nucleoside metabolic process	2.33E-04	1.40E-02
GO:0006364	rRNA processing	2.34E-04	1.40E-02
GO:0019222	regulation of metabolic process	3.02E-04	1.75E-02
GO:0055114	oxidation-reduction process	3.13E-04	1.78E-02
GO:0022607	cellular component assembly	3.60E-04	2.02E-02
GO:0016072	rRNA metabolic process	4.27E-04	2.35E-02
GO:0006796	phosphate-containing compound metabolic process	5.32E-04	2.87E-02
GO:0006793	phosphorus metabolic process	5.73E-04	3.04E-02
GO:0048518	positive regulation of biological process	6.66E-04	3.46E-02
GO:0009119	ribonucleoside metabolic process	6.78E-04	3.46E-02
GO:0019752	carboxylic acid metabolic process	7.09E-04	3.55E-02
GO:0043436	oxoacid metabolic process	8.96E-04	4.39E-02
GO:0006091	generation of precursor metabolites and energy	9.03E-04	4.39E-02
GO:0034613	cellular protein localization	9.95E-04	4.68E-02
GO:0042254	ribosome biogenesis	1.03E-03	4.74E-02
GO:0006629	lipid metabolic process	1.33E-03	5.99E-02
GO:0070727	cellular macromolecule localization	1.50E-03	6.60E-02
GO:0009167	purine ribonucleoside monophosphate metabolic process	1.77E-03	7.61E-02
GO:0044712	single-organism catabolic process	1.94E-03	8.15E-02
GO:0009126	purine nucleoside monophosphate metabolic process	1.98E-03	8.15E-02
GO:1902578	single-organism localization	2.63E-03	1.05E-01
GO:0044248	cellular catabolic process	3.48E-03	1.36E-01
GO:0009116	nucleoside metabolic process	3.64E-03	1.38E-01
GO:0031323	regulation of cellular metabolic process	4.27E-03	1.58E-01
GO:0009144	purine nucleoside triphosphate metabolic process	5.13E-03	1.85E-01
GO:0051641	cellular localization	5.24E-03	1.85E-01

GO:0046034	ATP metabolic process	5.34E-03	1.85E-01
GO:0009161	ribonucleoside monophosphate metabolic process	5.88E-03	1.94E-01
GO:0009205	purine ribonucleoside triphosphate metabolic process	5.89E-03	1.94E-01
GO:0080090	regulation of primary metabolic process	5.98E-03	1.94E-01
GO:0009141	nucleoside triphosphate metabolic process	7.24E-03	2.17E-01
GO:0044255	cellular lipid metabolic process	7.41E-03	2.17E-01
GO:0065003	macromolecular complex assembly	8.09E-03	2.27E-01
GO:0032787	monocarboxylic acid metabolic process	8.69E-03	2.35E-01
GO:0009123	nucleoside monophosphate metabolic process	9.22E-03	2.40E-01
GO:1901657	glycosyl compound metabolic process	9.91E-03	2.48E-01
GO:0009199	ribonucleoside triphosphate metabolic process	1.15E-02	2.76E-01
GO:0048522	positive regulation of cellular process	1.16E-02	2.76E-01
GO:0009117	nucleotide metabolic process	1.20E-02	2.76E-01
GO:0034248	regulation of cellular amide metabolic process	1.27E-02	2.76E-01
GO:0009150	purine ribonucleotide metabolic process	1.36E-02	2.76E-01
GO:0006950	response to stress	1.45E-02	2.76E-01
GO:0071822	protein complex subunit organization	1.83E-02	3.29E-01
GO:0009259	ribonucleotide metabolic process	1.85E-02	3.29E-01
GO:0060255	regulation of macromolecule metabolic process	1.91E-02	3.29E-01
GO:0033036	macromolecule localization	1.95E-02	3.29E-01
GO:0019693	ribose phosphate metabolic process	2.10E-02	3.29E-01
GO:0006753	nucleoside phosphate metabolic process	2.21E-02	3.29E-01
GO:0010608	posttranscriptional regulation of gene expression	2.61E-02	3.29E-01
GO:0034660	ncRNA metabolic process	2.88E-02	3.29E-01
GO:1901361	organic cyclic compound catabolic process	3.01E-02	3.29E-01
GO:0008610	lipid biosynthetic process	3.05E-02	3.29E-01

GO:0072521	purine-containing compound metabolic process	3.29E-02	3.29E-01
GO:1901700	response to oxygen-containing compound	3.42E-02	3.29E-01
GO:0055086	nucleobase-containing small molecule metabolic process	3.57E-02	3.29E-01
GO:0006163	purine nucleotide metabolic process	3.62E-02	3.29E-01
GO:0033365	protein localization to organelle	4.29E-02	3.29E-01
GO:0090304	nucleic acid metabolic process	4.75E-02	3.29E-01
GO:0044765	single-organism transport	4.91E-02	3.29E-01
GO:0071702	organic substance transport	4.99E-02	3.29E-01

Supplementary Table 3. Biological process enrichment results using differentially expressed genes between cluster 3 and cluster 1 samples.

GO ID	GO biological process complete	Enrichment P-value	FDR
GO:0071704	organic substance metabolic process	7.20E-27	1.43E-24
GO:0044238	primary metabolic process	8.50E-24	1.67E-21
GO:0044710	single-organism metabolic process	3.90E-22	7.64E-20
GO:0055114	oxidation-reduction process	8.00E-21	1.56E-18
GO:0009058	biosynthetic process	6.60E-15	1.28E-12
GO:0044711	single-organism biosynthetic process	2.30E-14	4.44E-12
GO:0044281	small molecule metabolic process	3.20E-14	6.14E-12
GO:0006807	nitrogen compound metabolic process	7.00E-14	1.34E-11
GO:1901576	organic substance biosynthetic process	1.10E-13	2.09E-11
GO:1901360	organic cyclic compound metabolic proces...	1.30E-13	2.46E-11
GO:0044260	cellular macromolecule metabolic process	2.10E-13	3.95E-11
GO:0006082	organic acid metabolic process	3.40E-13	6.36E-11
GO:0019752	carboxylic acid metabolic process	3.70E-13	6.88E-11
GO:0043436	oxoacid metabolic process	9.20E-13	1.70E-10
GO:0034641	cellular nitrogen compound metabolic pro...	3.50E-12	6.44E-10
GO:0010033	response to organic substance	4.30E-12	7.87E-10
GO:0044249	cellular biosynthetic process	7.90E-12	1.44E-09
GO:0051186	cofactor metabolic process	1.10E-11	1.99E-09
GO:1901700	response to oxygen-containing compound	1.50E-11	2.70E-09
GO:0015980	energy derivation by oxidation of organi...	5.40E-11	9.67E-09
GO:0043170	macromolecule metabolic process	6.50E-11	1.16E-08
GO:0006732	coenzyme metabolic process	1.10E-10	1.95E-08
GO:0046483	heterocycle metabolic process	1.50E-10	2.64E-08
GO:0006725	cellular aromatic compound metabolic pro...	2.20E-10	3.85E-08
GO:1901564	organonitrogen compound metabolic proces...	4.90E-10	8.53E-08
GO:0006091	generation of precursor metabolites and ...	1.00E-09	1.73E-07
GO:0044267	cellular protein metabolic process	1.20E-09	2.06E-07
GO:0044283	small molecule biosynthetic process	1.20E-09	2.06E-07
GO:0008610	lipid biosynthetic process	1.40E-09	2.38E-07
GO:0045333	cellular respiration	1.40E-09	2.38E-07
GO:0006629	lipid metabolic process	1.50E-09	2.52E-07
GO:0009725	response to hormone	3.20E-09	5.34E-07
GO:0001889	liver development	6.10E-09	1.01E-06
GO:0006139	nucleobase-containing compound metabolic...	7.40E-09	1.22E-06
GO:0031667	response to nutrient levels	1.00E-08	1.64E-06
GO:0061008	hepaticobiliary system development	1.20E-08	1.96E-06
GO:0009719	response to endogenous stimulus	1.40E-08	2.27E-06
GO:0014070	response to organic cyclic compound	1.40E-08	2.27E-06
GO:0010035	response to inorganic substance	2.40E-08	3.84E-06

GO:0043434	response to peptide hormone	3.50E-08	5.57E-06
GO:0010467	gene expression	4.00E-08	6.32E-06
GO:1901698	response to nitrogen compound	4.20E-08	6.59E-06
GO:0009991	response to extracellular stimulus	4.30E-08	6.71E-06
GO:0010243	response to organonitrogen compound	5.30E-08	8.22E-06
GO:0006396	RNA processing	5.90E-08	9.09E-06
GO:0042493	response to drug	1.00E-07	1.53E-05
GO:0006520	cellular amino acid metabolic process	1.70E-07	2.58E-05
GO:0019538	protein metabolic process	2.00E-07	3.02E-05
GO:0009056	catabolic process	2.10E-07	3.15E-05
GO:0006950	response to stress	2.30E-07	3.43E-05
GO:0019222	regulation of metabolic process	2.90E-07	4.29E-05
GO:0070887	cellular response to chemical stimulus	3.00E-07	4.41E-05
GO:1901652	response to peptide	3.80E-07	5.55E-05
GO:0032787	monocarboxylic acid metabolic process	4.00E-07	5.80E-05
GO:0008202	steroid metabolic process	4.50E-07	6.48E-05
GO:0051246	regulation of protein metabolic process	4.60E-07	6.58E-05
GO:0032870	cellular response to hormone stimulus	5.40E-07	7.67E-05
GO:1901605	alpha-amino acid metabolic process	6.80E-07	9.59E-05
GO:0044255	cellular lipid metabolic process	7.00E-07	9.80E-05
GO:0016053	organic acid biosynthetic process	7.40E-07	1.03E-04
GO:0046394	carboxylic acid biosynthetic process	7.40E-07	1.03E-04
GO:0043412	macromolecule modification	8.10E-07	1.11E-04
GO:0016126	sterol biosynthetic process	8.60E-07	1.17E-04
GO:0044248	cellular catabolic process	1.20E-06	1.62E-04
GO:0051188	cofactor biosynthetic process	1.20E-06	1.62E-04
GO:0006695	cholesterol biosynthetic process	1.30E-06	1.73E-04
GO:0022900	electron transport chain	1.40E-06	1.85E-04
GO:0080090	regulation of primary metabolic process	1.40E-06	1.85E-04
GO:0044085	cellular component biogenesis	1.50E-06	1.95E-04
GO:0034470	ncRNA processing	1.60E-06	2.06E-04
GO:0048511	rhythmic process	1.60E-06	2.06E-04
GO:0071310	cellular response to organic substance	1.60E-06	2.06E-04
GO:0033554	cellular response to stress	1.70E-06	2.14E-04
GO:0043648	dicarboxylic acid metabolic process	1.80E-06	2.25E-04
GO:1901701	cellular response to oxygen-containing c...	1.90E-06	2.36E-04
GO:0034660	ncRNA metabolic process	2.10E-06	2.58E-04
GO:0006464	cellular protein modification process	2.40E-06	2.93E-04
GO:0006793	phosphorus metabolic process	2.40E-06	2.93E-04
GO:0036211	protein modification process	2.40E-06	2.93E-04
GO:1901575	organic substance catabolic process	2.40E-06	2.93E-04
GO:0022904	respiratory electron transport chain	2.70E-06	3.19E-04
GO:0034645	cellular macromolecule biosynthetic proc...	3.00E-06	3.51E-04
GO:0007584	response to nutrient	3.20E-06	3.71E-04

GO:0048732	gland development	3.30E-06	3.80E-04
GO:0009059	macromolecule biosynthetic process	3.50E-06	3.99E-04
GO:0065003	macromolecular complex assembly	3.50E-06	3.99E-04
GO:0006979	response to oxidative stress	3.60E-06	4.03E-04
GO:0033993	response to lipid	3.70E-06	4.11E-04
GO:0048545	response to steroid hormone	5.60E-06	6.16E-04
GO:0032922	circadian regulation of gene expression	5.90E-06	6.43E-04
GO:0032268	regulation of cellular protein metabolic...	6.20E-06	6.70E-04
GO:0010038	response to metal ion	6.40E-06	6.85E-04
GO:0009060	aerobic respiration	6.70E-06	7.10E-04
GO:1990267	response to transition metal nanoparticl...	9.50E-06	9.98E-04
GO:0090304	nucleic acid metabolic process	9.60E-06	9.98E-04
GO:0044282	small molecule catabolic process	1.00E-05	1.03E-03
GO:0032868	response to insulin	1.20E-05	1.22E-03
GO:0048518	positive regulation of biological proces...	1.20E-05	1.22E-03
GO:0006796	phosphate-containing compound metabolic ...	1.30E-05	1.30E-03
GO:0031669	cellular response to nutrient levels	1.70E-05	1.68E-03
GO:1901615	organic hydroxy compound metabolic proce...	1.80E-05	1.76E-03
GO:0006066	alcohol metabolic process	1.90E-05	1.84E-03
GO:0051791	medium-chain fatty acid metabolic proces...	2.00E-05	1.92E-03
GO:0031323	regulation of cellular metabolic process	2.10E-05	2.00E-03
GO:0042594	response to starvation	2.30E-05	2.16E-03
GO:0016125	sterol metabolic process	2.40E-05	2.23E-03
GO:0044712	single-organism catabolic process	2.40E-05	2.23E-03
GO:0022607	cellular component assembly	2.50E-05	2.28E-03
GO:0006631	fatty acid metabolic process	2.60E-05	2.34E-03
GO:2000323	negative regulation of glucocorticoid re...	2.60E-05	2.34E-03
GO:0031100	organ regeneration	2.70E-05	2.38E-03
GO:0009605	response to external stimulus	2.80E-05	2.44E-03
GO:0016051	carbohydrate biosynthetic process	2.80E-05	2.44E-03
GO:0006790	sulfur compound metabolic process	2.90E-05	2.47E-03
GO:0032869	cellular response to insulin stimulus	3.10E-05	2.60E-03
GO:0008203	cholesterol metabolic process	3.20E-05	2.66E-03
GO:0006733	oxidoreduction coenzyme metabolic proces...	3.30E-05	2.71E-03
GO:0060255	regulation of macromolecule metabolic pr...	3.40E-05	2.75E-03
GO:0007623	circadian rhythm	3.70E-05	2.96E-03
GO:0006270	DNA replication initiation	3.90E-05	3.08E-03
GO:0009108	coenzyme biosynthetic process	4.10E-05	3.20E-03
GO:0009119	ribonucleoside metabolic process	4.30E-05	3.31E-03
GO:0031324	negative regulation of cellular metaboli...	4.30E-05	3.31E-03
GO:0048523	negative regulation of cellular process	4.40E-05	3.31E-03
GO:0042752	regulation of circadian rhythm	4.60E-05	3.40E-03
GO:0048522	positive regulation of cellular process	4.70E-05	3.43E-03
GO:0006694	steroid biosynthetic process	4.90E-05	3.53E-03

GO:0009267	cellular response to starvation	4.90E-05	3.53E-03
GO:0009892	negative regulation of metabolic process	5.10E-05	3.57E-03
GO:0016072	rRNA metabolic process	5.10E-05	3.57E-03
GO:0046165	alcohol biosynthetic process	5.40E-05	3.67E-03
GO:1901657	glycosyl compound metabolic process	5.40E-05	3.67E-03
GO:0071702	organic substance transport	5.50E-05	3.67E-03
GO:0009116	nucleoside metabolic process	6.10E-05	3.97E-03
GO:0043933	macromolecular complex subunit organizat...	6.10E-05	3.97E-03
GO:0009628	response to abiotic stimulus	6.30E-05	3.97E-03
GO:0010605	negative regulation of macromolecule met...	6.50E-05	4.03E-03
GO:0051248	negative regulation of protein metabolic...	6.60E-05	4.03E-03
GO:0006412	translation	6.90E-05	4.14E-03
GO:0006575	cellular modified amino acid metabolic p...	7.10E-05	4.19E-03
GO:0034622	cellular macromolecular complex assembly	7.40E-05	4.29E-03
GO:0032269	negative regulation of cellular protein ...	7.50E-05	4.29E-03
GO:0006364	rRNA processing	7.90E-05	4.42E-03
GO:0043254	regulation of protein complex assembly	8.10E-05	4.46E-03
GO:0048519	negative regulation of biological proces...	8.20E-05	4.46E-03
GO:0046890	regulation of lipid biosynthetic process	8.30E-05	4.46E-03
GO:0097190	apoptotic signaling pathway	8.30E-05	4.46E-03
GO:0070482	response to oxygen levels	9.10E-05	4.64E-03
GO:0071407	cellular response to organic cyclic comp...	9.20E-05	4.64E-03
GO:0031668	cellular response to extracellular stimu...	9.70E-05	4.75E-03
GO:0043153	entrainment of circadian clock by photop...	9.70E-05	4.75E-03
GO:0007568	aging	9.90E-05	4.75E-03
GO:0007005	mitochondrion organization	1.10E-04	5.06E-03
GO:0009649	entrainment of circadian clock	1.10E-04	5.06E-03
GO:0019362	pyridine nucleotide metabolic process	1.10E-04	5.06E-03
GO:0046496	nicotinamide nucleotide metabolic proces...	1.10E-04	5.06E-03
GO:0071375	cellular response to peptide hormone sti...	1.10E-04	5.06E-03
GO:0072524	pyridine-containing compound metabolic p...	1.10E-04	5.06E-03
GO:0046128	purine ribonucleoside metabolic process	1.20E-04	5.06E-03
GO:0071383	cellular response to steroid hormone sti...	1.20E-04	5.06E-03
GO:0071495	cellular response to endogenous stimulus	1.20E-04	5.06E-03
GO:1901566	organonitrogen compound biosynthetic pro...	1.20E-04	5.06E-03
GO:0042558	pteridine-containing compound metabolic ...	1.30E-04	5.06E-03
GO:0016070	RNA metabolic process	1.40E-04	5.06E-03
GO:0019637	organophosphate metabolic process	1.40E-04	5.06E-03
GO:0070271	protein complex biogenesis	1.40E-04	5.06E-03
GO:0097305	response to alcohol	1.40E-04	5.06E-03
GO:0010608	posttranscriptional regulation of gene e...	1.50E-04	5.06E-03
GO:0042278	purine nucleoside metabolic process	1.50E-04	5.06E-03
GO:0019216	regulation of lipid metabolic process	1.60E-04	5.06E-03
GO:0031960	response to corticosteroid	1.60E-04	5.06E-03

GO:0033197	response to vitamin E	1.60E-04	5.06E-03
GO:0006461	protein complex assembly	1.70E-04	5.06E-03
GO:0009893	positive regulation of metabolic process	1.70E-04	5.06E-03
GO:0010604	positive regulation of macromolecule met...	1.70E-04	5.06E-03
GO:0050790	regulation of catalytic activity	1.70E-04	5.06E-03
GO:0006886	intracellular protein transport	1.80E-04	5.06E-03
GO:0051234	establishment of localization	1.80E-04	5.06E-03
GO:0046184	aldehyde biosynthetic process	1.90E-04	5.06E-03
GO:1901653	cellular response to peptide	1.90E-04	5.06E-03
GO:0031325	positive regulation of cellular metaboli...	2.10E-04	5.06E-03
GO:0045184	establishment of protein localization	2.10E-04	5.06E-03
GO:0051384	response to glucocorticoid	2.10E-04	5.06E-03
GO:0034614	cellular response to reactive oxygen spe...	2.30E-04	5.06E-03
GO:0043489	RNA stabilization	2.40E-04	5.06E-03
GO:0048255	mRNA stabilization	2.40E-04	5.06E-03
GO:0022613	ribonucleoprotein complex biogenesis	2.50E-04	5.06E-03
GO:0046907	intracellular transport	2.50E-04	5.06E-03
GO:0006810	transport	2.60E-04	5.06E-03
GO:0046685	response to arsenic-containing substance	2.60E-04	5.06E-03
GO:0070647	protein modification by small protein co...	2.60E-04	5.06E-03
GO:0009161	ribonucleoside monophosphate metabolic p...	2.70E-04	5.06E-03
GO:0065009	regulation of molecular function	2.70E-04	5.06E-03
GO:0015031	protein transport	2.80E-04	5.06E-03
GO:0000377	RNA splicing, via transesterification re...	2.90E-04	5.06E-03
GO:0000398	mRNA splicing, via spliceosome	2.90E-04	5.06E-03
GO:0005978	glycogen biosynthetic process	3.00E-04	5.06E-03
GO:0009250	glucan biosynthetic process	3.00E-04	5.06E-03

Supplementary Table 4. Network results obatined based on IPA platform.

ID	Score	Focus Molecules	Diseases & Functions	Molecules in Network
1	34	33	Cancer, Cell Death and Survival, Organismal Injury and Abnormalities	60S ribosomal subunit,ABCF1,ADARB1,BOP1, C7orf50,CEP250,CNBP,DDX24,DDX56,GKAP1, HNRNPU,IFRD2,NOC4L,p85 (pik3r),PIN4,PINX1, PLEKHJ1,RPL4,RPL15,RPL19,RPL21,RPL26,RPL28,RPL31,RPL36,RPL10A,RPL18A,RPL23A,RPL27A,RPL36AL, RPLP1,Rps3a1,Rrbp1,SRP14,THAP7
2	34	33	Molecular Transport, RNA Trafficking, Carbohydrate Metabolism	ADAR,AGTR1,ALYREF,ANGEL2,B9D2,C14orf166,C9orf142,ERGIC1,Fus,GATC,GPAA1,HNRNPA0,HNRNPA1, Hnrnpa3,HNRNPH2,HNRNPM,HNRPA1-HNRPA2B1-POT1-TERF1-TERF2,HSD11B2,MAST3,MATR3,MRPS10, Nuclear factor 1, NUDT16L1,PIGS,PIGT,PIGU,PKP2,PURB,RAVER1,RPA3,SUPV3L1,THOC2,TMEM11,TMEM131,ZNF638
3	34	33	Cell Morphology, Cellular Assembly and Organization, Cellular Function and Maintenance	AKR7A2,ATG4D,C12orf43,Cdc42,CMC2,DDA1,ECHS1,FARSA,FARSB,FLCN,FNIP1,GABARAPL1,GABARAPL2, HDL-cholesterol,LAMP1,LAMP2,MCC,Mir122a,b,MMAB,MVK,NAGLU,NANS,PAICS,PCSK9,PRKCSH,RSBN1L, SGPL1,SLC25A10,SRM,TBCEL,TERF1,TFEB,TKT,TMX1,TRMT1
4	34	33	Cellular Assembly and Organization, Developmental Disorder, Neurological Disease	ACTB,ARMC5,CCT3,Creb,DBN1,DCTN5,DSP,DYNLL1,FLNB,FOXA3,GFM2,HGH1,KLHL17,KRT8,KRT18, MESP2,PLEC,POLE,PPFIBP1,PPL,PPP1CB,PROSER2,QRSL1,RPS6KA,SIX5,SLC2A2,SPTAN1,SYNPO,TMEM201, TRHDE,TRIM6-TRIM34,TUBB,TUBB2A,TUBG1,UBAP2
5	34	33	Developmental Disorder, Embryonic Development, Organismal Development	BRI3,CCDC86,CEBPA,CRELD2,CSNK2B,CYCS,EMG1,FABP5,FAM133B,FOXA2,FRG1,HECW2,IKBKE,LMNA, LYSMD3,MGP,MIB1,MRPL54,NADPH oxidase,NARF,NCL,NPM1,PRKAR1A,PRMT5,Pro-inflammatory Cytokine, RCL1,SBNO1,SIPA1L2,SLC25A42,SPSB3,TBC1D23,TFG,TMEM64,ZFP62,ZNRF2
6	31	32	Cellular Assembly and Organization, Cancer, Neurological Disease	AP-3,apyrase,ASAP2,ASB3/GPR75-ASB3,BCCIP,Calbindin,CANT1,CENPV,COQ8A,DDX41,DNASE1,DPM1, FAM46A,FYN,GINM1,HIGD1A,M6PR,MON1A,MPEG1,MRPS16,NUBP2,PLIN3,REEP6,RIN3,SAMD1,SLC30A3, STX17,TBRG4,TMEM159,TMEM263,TNS2,TUBE1,VAMP7,VPS41,VPS33A
7	31	32	Connective Tissue Disorders, Dermatological Diseases and Conditions, Drug Metabolism	BCL7C,CIC,CNOT2,CNOT9,CNOT10,CNOT6L,CUTA,DCAF8,DNA-directed DNA polymerase,FIGN,GLTSCR1, GRB2,GTPBP3,HIBCH,LANCL1,Mhc2 Alpha,MRPS12,MRPS15,MSTO1,MYRIP,NCKAP5,NFYC, phosphatidylinositol-4,5-bisphosphate 3-kinase,POLD1,POP5,RPP25,RPP38,SEC62,SGMS2,SHKBP1,SKAP2, SLCO2A1,SMARCD2,TBC1D9B,TESK1

8	31	32	Cell Signaling, Dental Disease, Dermatological Diseases and Conditions	BAIAP2L1,C1orf174,CAMSAP3,CMPK1,COPS6,DISP1,EHBP1,EID1,ERBB,FBXO21,GRWD1,KLHL9,KLHL22, METTL1,METTL18,MRPS2,MRPS11,NAB2,NDPK,NME7,PCMTD2,PDLIM5,PHF14,RHOBTB1,ROGDI,RSF1, SCAF11,SLIRP,SRC (family),SRP68,TLE2,TTC19,TUBGCP6,UBAC1,WDR4
9	31	32	Cancer, Cell Death and Survival, Cell-To-Cell Signaling and Interaction	CDC34,CHPF,DTX3L,EXOSC5,EXOSC6,GADD45GIP1,H2AFV,JADE1,MRPL4,MRPL34,MRPL37,MRPL38, MRPL42,MRPL55,MRPS18B,NOL6,PARP14,PLC,RMND5A,RNF103,RNF146,RNF144A,RPUSD3,RRP7A, Scf skp2,SECISBP2L,TATDN2,TBL3,TNFRSF11B,TSPAN12,TTI1,UBE2,UBE2H,UBE2V1,UPF2
10	31	32	Cardiovascular Disease, Developmental Disorder, Hematological Disease	AHSA1,APC/APC2,DCUN1D1,FBXO3,GPS1,Importin beta,JMJD8,KLHL26,MLLT1,MRPL12,NOL10,PHIP, PPM1G,PROS1,RGS3,Ribosomal 40s subunit,RPS2,RPS10,RPS19,RPS21,RPS29,RPS15A,RPS3A,RPSA,S1PR1, SPATA13,SYPL1,TIAL1,TSR1,TXNDC5,VARS,ZBTB1,ZNF358,ZNF777,ZUFSP
11	31	32	Cellular Assembly and Organization, Lipid Metabolism, Small Molecule Biochemistry	ACOT4,ACOT7,ACOT8,ACOT13,acyl-CoA hydrolase,AIM1L,AMACR,BAAT,BSCL2,CCPG1,CIDEC,DDX49, GPD1,HAO1,HAO2,HELZ2,Hsd3b4 (includes others),JMJD1C,LCN12,MED15,MED16,MED27,MED13L,NOCT, NUDT19,palmitoyl-CoA hydrolase,PEX5,PPARG,RARRES2,RNF144B,Sik1,Slc25a1,THRAP3,TMED3,TRAP/Media
12	31	32	Amino Acid Metabolism, Small Molecule Biochemistry, Drug Metabolism	ACYP1,AHCY,AK3,BABAM1,BCKDHA,BCKDK,carboxy-lyase,COMT,DAPK3,DOLPP1,ECD,ECHDC1,GADD45, GOT1,GPT2,GPX1,GPX3,Hepatic Transaminase,KDM5A,MBTD1,MFSD2A,Mt1m/Mt2A,NR3C1,PPM1K,SAP30BP, SELENBP1,SLC43A1,SPPL2B,SRSF9,TM2D2,TMEM120A,Uox,URAD,WBSCR22,ZNF48
13	31	32	DNA Replication, Recombination, and Repair, Gene Expression, Developmental Disorder	ARL5A,CBX1,CBX3,CCDC174,CHAF1B,CHMP3,EML5,GCNT2,GTF2A1,Histone h3,HP1,IPO4,JADE2,KDM3A, KDM5B,KLF11,METTL9,MIS12,PLBD2,PTCD2,RAB38,RABL6,RBM47,RPRM,RTN4RL2,SBF1,SETDB1,SPC24, TMEM79,Top2,TTLL12,UHRF1,ZBTB44,ZNF445,ZWINT
14	31	32	Lipid Metabolism, Small Molecule Biochemistry, Digestive System Development and Function	ABHD6,AES,ARHGEF39,ARPP19,CDC23,CEBPB,CEP44,CIRBP,CYP17A1,DGKZ,DHCR7,DHCR24,DUB, HERPUD1,IBTK,Igh (family),LDL-cholesterol,LDLR,MSR1,MVP,NIF3L1,NR2C2,NUDT22,PPP1R16A,RBPMS, SAT1,SEMA4G,SERINC1,SOX5,TMEM50B,TRIB3,UFL1,URB1,USP10,USP32
15	30	31	Post-Translational Modification, Molecular Transport, Cancer	AUP1,C5orf24,CARD6,CITED4,Cyb5r3,DAD1,DDOST,DOHH,EMC6,EMC7,EMC9,FASTKD1,Gpd,HGFAC,Ktn1, MAGT1,malate dehydrogenase,MCOLN1,MMGT1,NFkB (complex),NOP14,OSBPL3,OTULIN,PCNP,PPARα-RXRα, RNF25,RNF141,RPN2,RWDD3,SCLY,SLC2A5,ST3GAL1,TMEM57,TMEM14C,YIF1A

16	30	31	Behavior, Nervous System Development and Function, Lipid Metabolism	ALAS1,ARNTL,CLOCK,CRY1,CRY2,Cryptochrome,DARS,DBP,F9,FBXL3,HLF,HNF4G,IARS,IPMK,JUN,MTHFR, NPAS2,NR1D1,NRAP,PDXK,PER1,PER2,PER3,PERIOD,PNRC2,PRELID3B,RNF187,SCNN1A,SLC6A6,Snrpc,Tcf 1/3/4, TEF,TIMELESS,UBIQUITIN LIGASE,USP2
17	30	31	RNA Post-Transcriptional Modification, Cancer, Cellular Development	aldehyde dehydrogenase (NAD),ALDH1A3,ALDH1L2,ALDH3A2,ALDH3B1,ALDH7A1,ALG3,Alpha 1 antitrypsin, ARID4A,ATL3,C17orf96,C7orf26,CCDC59,chymotrypsin,DALRD3,DEDD,EDEM1,FKBP14,GDE1,Gsk3,HOXB3,INTS3, INTS5,INTS6,IPO13,KALRN,PAM,PNISR,Rpl23a,SERPINA4,SERPINA6,SUDS3,TMEM25,UHMK1,ZCCHC6
18	30	31	RNA Post-Transcriptional Modification, Cell Cycle, DNA Replication, Recombination, and Repair	AFMID,CHERP,DSE,EFTUD2,GPN2,histone deacetylase,HNRNPA2B1,HTATSF1,KDM3B,LSM2,LSM12,MPND, MTORC1,PMVK,PQBP1,PRPF19,PSPC1,RBM42,RNA polymerase iii,RPAP1,SF3A1,Sf3a2,SF3B4,SF3B5,snRNP, SNRPA,SNRPC,SNRPF,SPAG8,SSB,TTC14,TXNL4A,USP39,WBP4,ZNF76
19	30	31	Developmental Disorder, Hereditary Disorder, Metabolic Disease	Ap1,ATP6AP2,COMMD9,CRBN,DHDDS,ECSIT,Hat,HNRNPK,LITAF,METTL5,MT-ND1,MT-ND2,MT-ND4,MT-ND5, NDUFA7,NDUFA11,NDUFV2,PRMT3,PRMT7,PUF60,RAB4,RAB5C,RABEP1,RABGEF1,RANBP1,RANGAP1,REN, Ryr,ST8SIA1,TBL2,THRA,TMUB1,TOM1,USPL1,UTRN
20	30	31	Cell Signaling, Cellular Function and Maintenance, Molecular Transport	ABCA1,ABCC4,ABCC9,ACD,AOX1,ARHGAP42,ATPase,CD3,CRNKL1,DDT,EIF4A2,ERICH5,FYTTD1,KCNJ8, KSR2,MYO19,NAMPT,ORAI1,P glycoprotein,PAFAH1B3,PCSK7,PCYT2,PEX6,PPRC1,PREB,Rsrc2,Sphk,SRSF2, SRSF4,SRSF7,STIM2,TMX3,TOR1B,ZC3H11A,ZFP36L1
21	30	31	Dermatological Diseases and Conditions, Organismal Injury and Abnormalities, Respiratory System Development and Function	AHR,BMP2,BSG,CAPN12,CDS2,COL1A1,Collagen type I,CRK,DACT2,EPAS1,ETS1,Fgf,FMO2,GJA1,GPD1L,HGS, Histone h4,Integrin,KDR,LOC100365810/Rps17,MYBBP1A,NAA60,Nat8f5,NCLN,NFYB,NPEPL1,Pcp4l1,SERPINB6, SERPINF1,SLC16A4,SLC25A28,SLC25A30,TGFBR2,TMEM218,ZNF524
22	28	30	Lipid Metabolism, Small Molecule Biochemistry, Neurological Disease	ABLIM3,ACSM3,Anti-inflammatory Cytokine,AQP11,B4GALNT1,CFI,FETUB,Inhibin,LTBP4,Masp1,NAT6,NNMT, PMM1,PMM2,RBM19,RBMS1,Ripply1,SBNO2,SLC39A8,SLC5A2,SPARCL1,SPIDR,Srp30,ST3GAL5,STARD10,TCN2, TGFB1,TMEM259,TMEM184B,TTLL3,TWIST,Type II Receptor,VASN,WFIKKN1,ZNF354A
23	28	30	Developmental Disorder, Hereditary Disorder, Metabolic Disease	2-oxoglutarate:oxygen oxidoreductase,ALKBH1,ALKBH8,CD274,chemokine,COG7,COG8,CYC1,EGLN,EGLN1, EXO1,FASTK,FKBP8,FOXH1,IL17RB,INTERLEUKIN,MT-CYB,MYEF2,OAT,PEX19,PGLYRP2,POTEE/POTEF, PXMP4,RNase A,SIGIRR,SLC37A4,SSSCA1,TRAF3IP3,TYW5,UQCR10,UQCRC1,UQCRC2,UQCRQ,YARS,ZNF281

24	28	30	Gene Expression, Cellular Development, Cellular Growth and Proliferation	AEBP2,BCL3,BMP7,CCNG2,CREBBP,FBXW8,GPBP1,Hdac,HISTONE,histone-lysine N-methyltransferase,ID2,ID3, Igg3,IL6R,IRF6,KAT2A,KAT2B,KLF6,KMT5A,KMT5B,mir-22,MKNK2,Nat8f3 (includes others),NTMT1,POLRMT, Ppp2c,RTP4,SETD3,SLC50A1,SMAD1,SMG5,SYMPK,UNG,WDR46,ZFPM1
25	28	30	Infectious Diseases, Carbohydrate Metabolism, Cellular Function and Maintenance	26s Proteasome,AIFM1,ALAD,ALDH6A1,BICDL1,DCTN1,ERBB3,FAM8A1,FASN,GFRA3,HMGCR,INSR,MGEA5, MRPS26,Mug1 (includes others),MVD,NDUFB9,NFKBIB,NOL12,PARP,PGRMC2,PI3K (family),SEC24B,SLC35C2, SPATA2L,sphingomyelinase,SUCLA2,SYVN1,TBCE,TSPAN4,TUBA4A,TXNIP,Ubiquitin,UCP2,YBX1
